# Supplementary material for: Spatiotemporal single-cell regulatory atlas reveals neural crest lineage diversification and cellular function during tooth morphogenesis
Source: Nat Commun. 2022 Aug 16;13:4803. doi: 10.1038/s41467-022-32490-y (PMC9381504; doi:10.1038/s41467-022-32490-y)
Supplement: Supplementary file 1 — Supplementary Information [file 41467_2022_32490_MOESM1_ESM.pdf]

Spatiotemporal single-cell regulatory atlas reveals neural crest lineage diversification and cellular function during tooth morphogenesis

This document includes:

Supplementary Fig. 1 to Supplementary Fig. 12

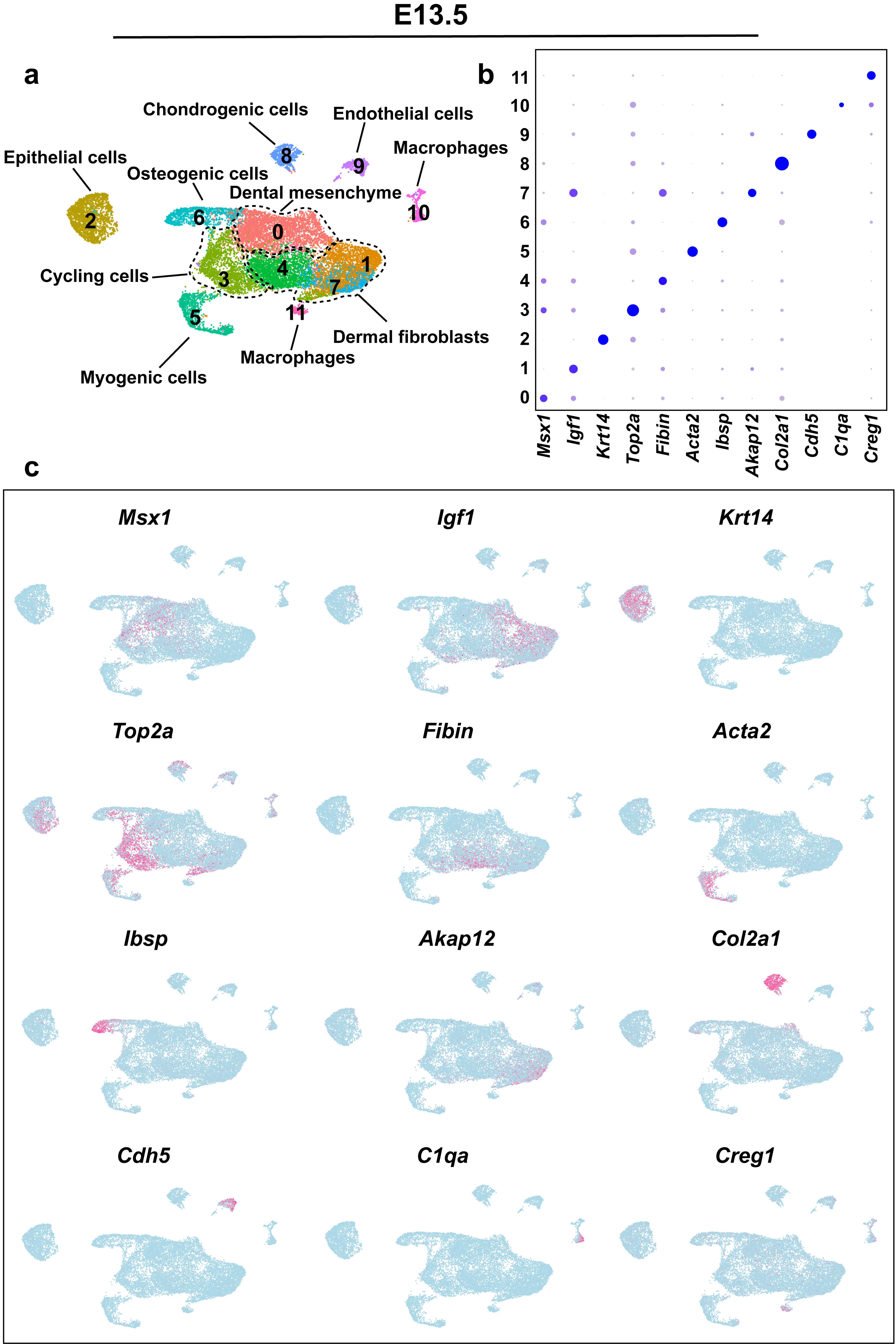

E13.5
a
Chondrogenic cells
Endothelial cells
Epithelial cells
Macrophages
Osteogenic cells
Dental mesenchyme
Cycling cells
Dermal fibroblasts
Macrophages
Myogenic cells
b
Msx1
Igf1
Krt14
Top2a
Fibin
Acta2
Ibsp
Akap12
Col2a1
Cdh5
C1qa
Creg1
c
Msx1
Igf1
Krt14
Top2a
Fibin
Acta2
Ibsp
Akap12
Col2a1
Cdh5
C1qa
Creg1

Supplementary Fig. 1 Cell populations in the mouse molar and its surrounding tissue at E13.5. a UMAP plot of cell types in the mouse molar and its surrounding tissue at E13.5. Cell types were annotated based on their expression of cell type-specific marker genes; **b** Dotplot of signature genes in the cell populations shown in **a**. **c** Feature plot of markers genes shown in **b**.

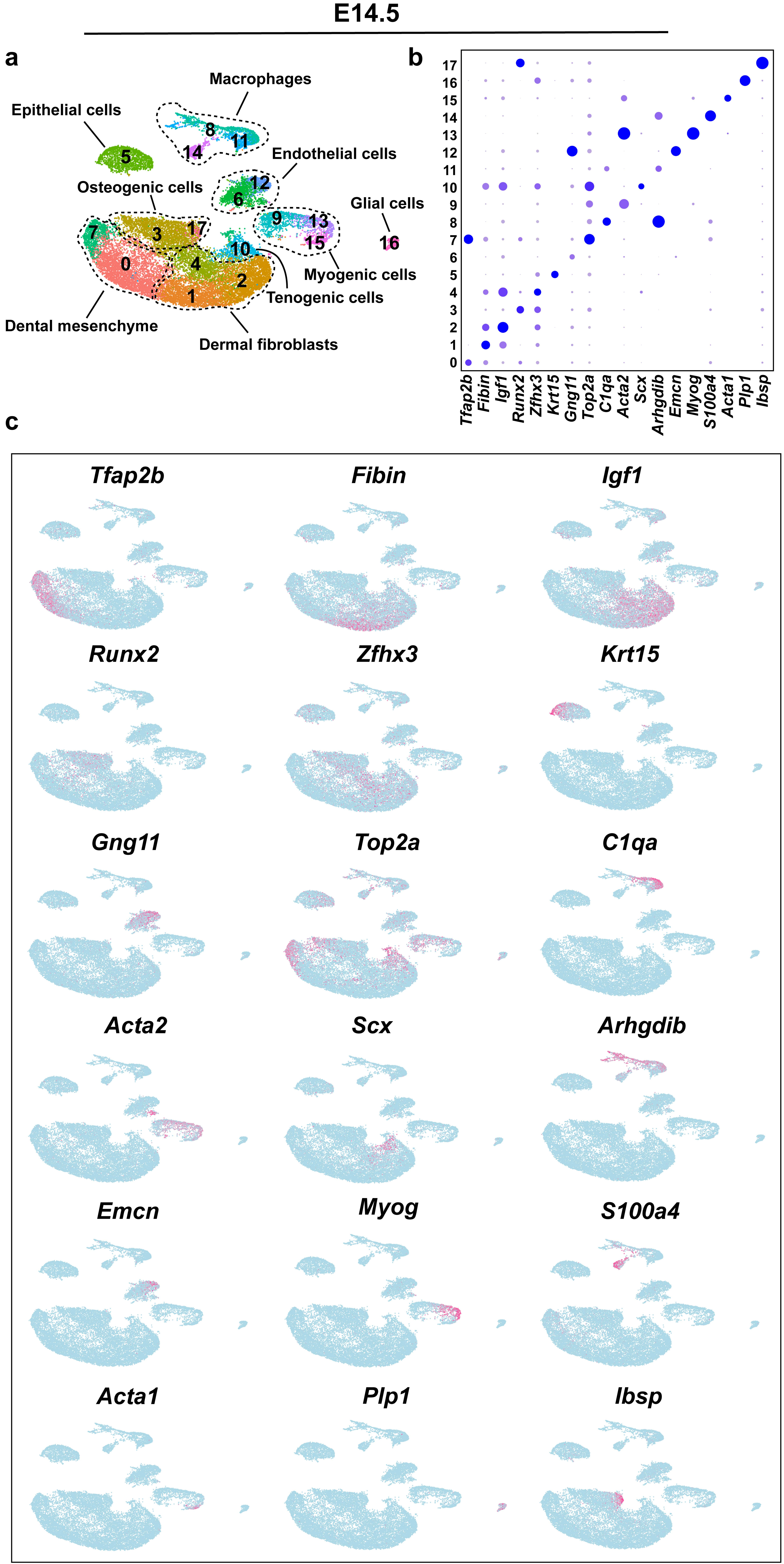

E14.5
a
Macrophages
Epithelial cells
Endothelial cells
Osteogenic cells
Glial cells
Myogenic cells
Tenogenic cells
Dental mesenchyme
Dermal fibroblasts
0
1
2
3
4
5
6
7
8
9
10
11
12
13
14
15
16
17
b
Tfap2b
Fibin
Igf1
Runx2
Zfhx3
Krt15
Gng11
Top2a
C1qa
Acta2
Scx
Arhgdib
Emcn
Myog
S100a4
Acta1
Plp1
Ibsp
c
Tfap2b
Fibin
Igf1
Runx2
Zfhx3
Krt15
Gng11
Top2a
C1qa
Acta2
Scx
Arhgdib
Emcn
Myog
S100a4
Acta1
Plp1
Ibsp

Supplementary Fig. 2 Cell populations in the mouse molar and its surrounding tissue at E14.5. **a** UMAP plot of cell types in the mouse molar and its surrounding tissue at E14.5. **b** Dotplot of signature genes in the cell populations shown in **a**. **c** Feature plot of markers genes shown in **b**.

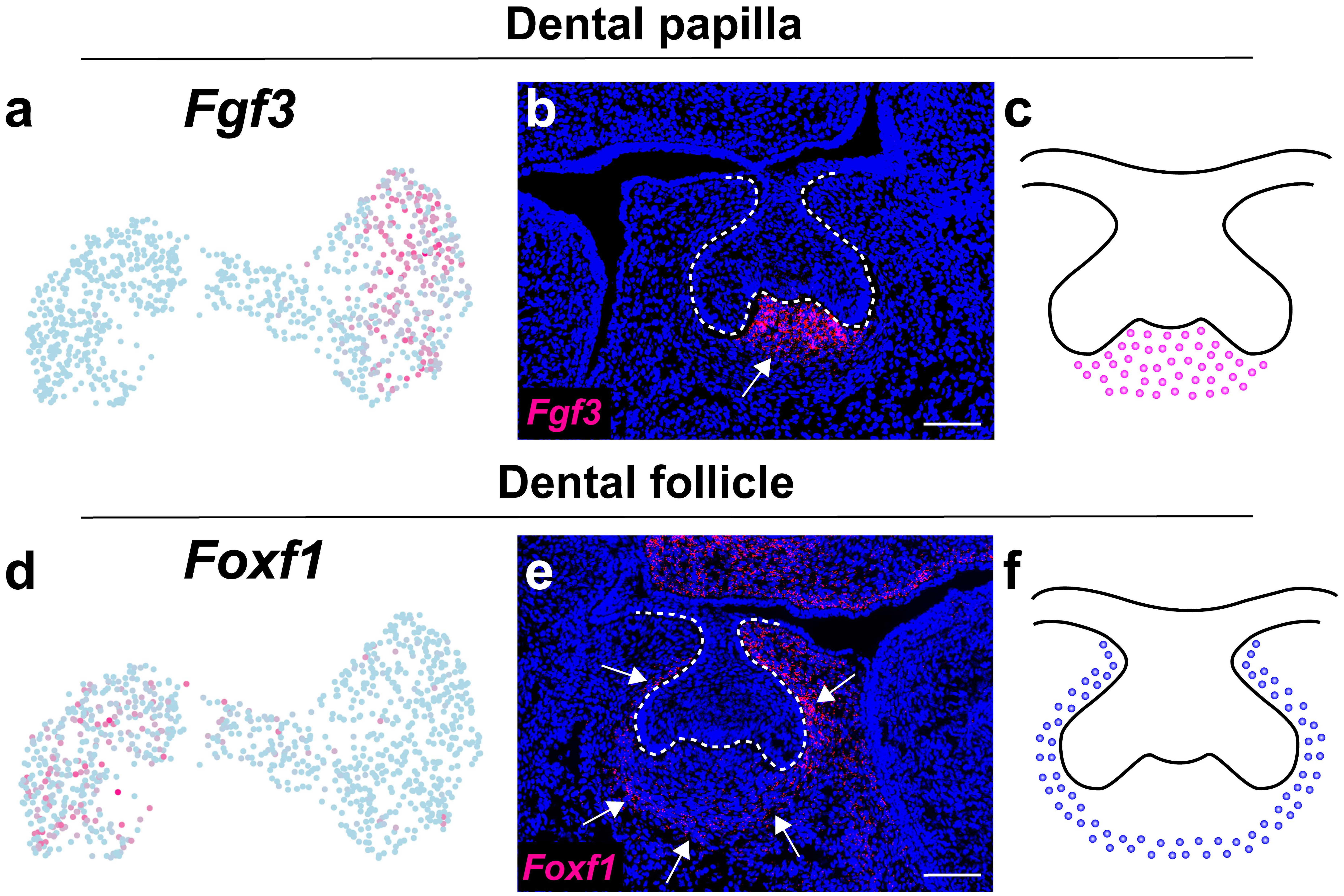

Dental papilla
a
Fgf3
b
Fgf3
c
Dental follicle
d
Foxf1
e
Foxf1
f

Supplementary Fig. 3 Cellular domain of the dental mesenchyme at E14.5. a, **b** Feature plot and RNAscope staining of *Fgf3* in the dental papilla (n=3). **c** Cellular domain of the dental papilla in the mouse molar at E14.5. **d**, **e** Feature plot and RNAscope staining of *Foxf1* in the dental follicle (n=3). **f** Cellular domain of the dental follicle in the mouse molar at E14.5. White dotted lines outline the dental epithelial cells in the mouse molar. White arrows point to the positive signals. Scale bars, 100μm.

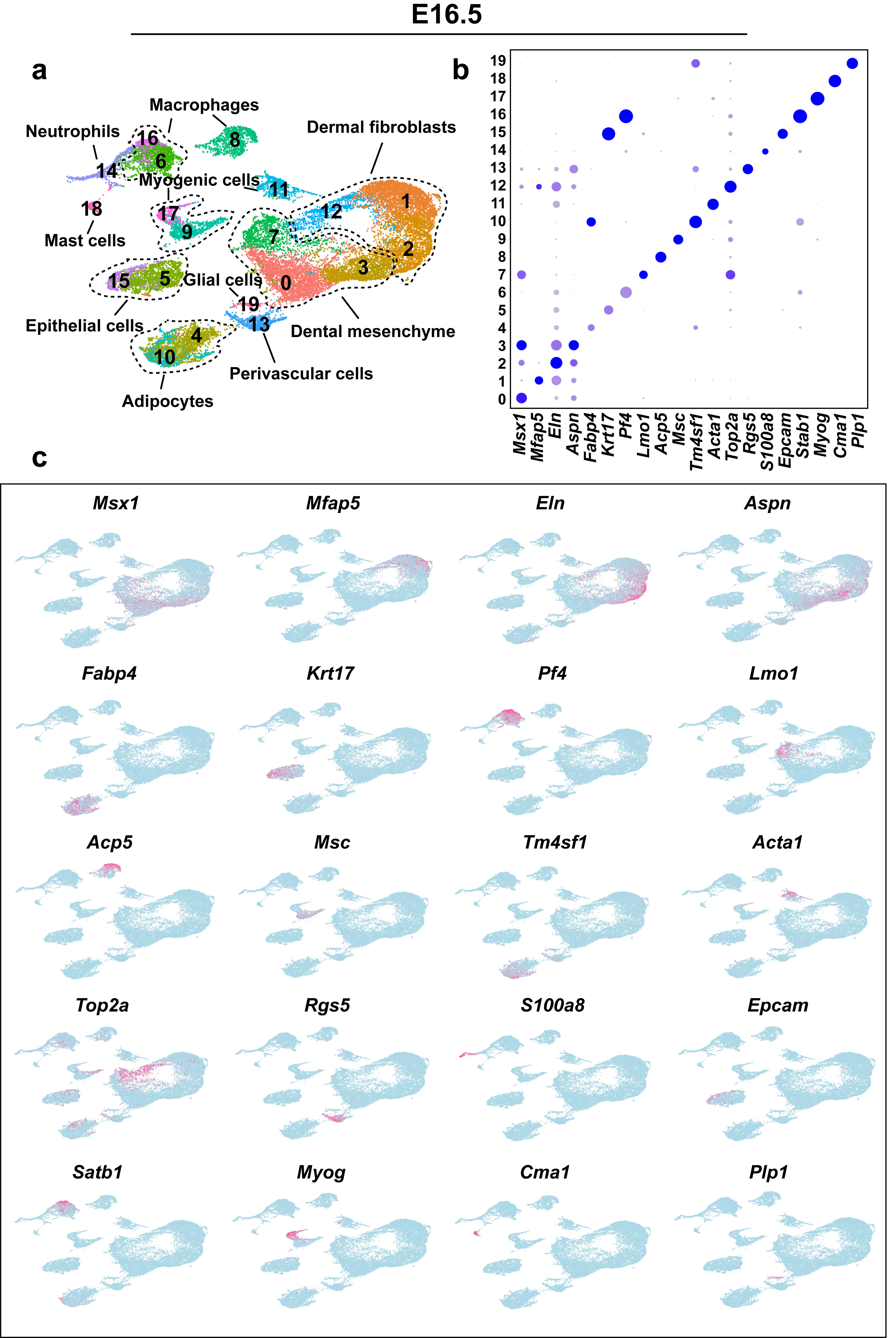

E16.5
a
Macrophages
Neutrophils
Dermal fibroblasts
Myogenic cells
Mast cells
Glial cells
Epithelial cells
Dental mesenchyme
Perivascular cells
Adipocytes
b
Msx1
Mfap5
Eln
Aspn
Fabp4
Krt17
Pf4
Lmo1
Acp5
Msc
Tm4sf1
Acta1
Top2a
Rgs5
S100a8
Epcam
Stab1
Myog
Cma1
Plp1
c
Msx1
Mfap5
Eln
Aspn
Fabp4
Krt17
Pf4
Lmo1
Acp5
Msc
Tm4sf1
Acta1
Top2a
Rgs5
S100a8
Epcam
Satb1
Myog
Cma1
Plp1

Supplementary Fig. 4 Cell populations in the mouse molar and its surrounding tissue at E16.5. **a** UMAP plot of cell types in the mouse molar and its surrounding tissue at E16.5. **b** Dotplot of signature genes in the cell populations shown in **a**. **c** Feature plot of markers genes shown in **b**.

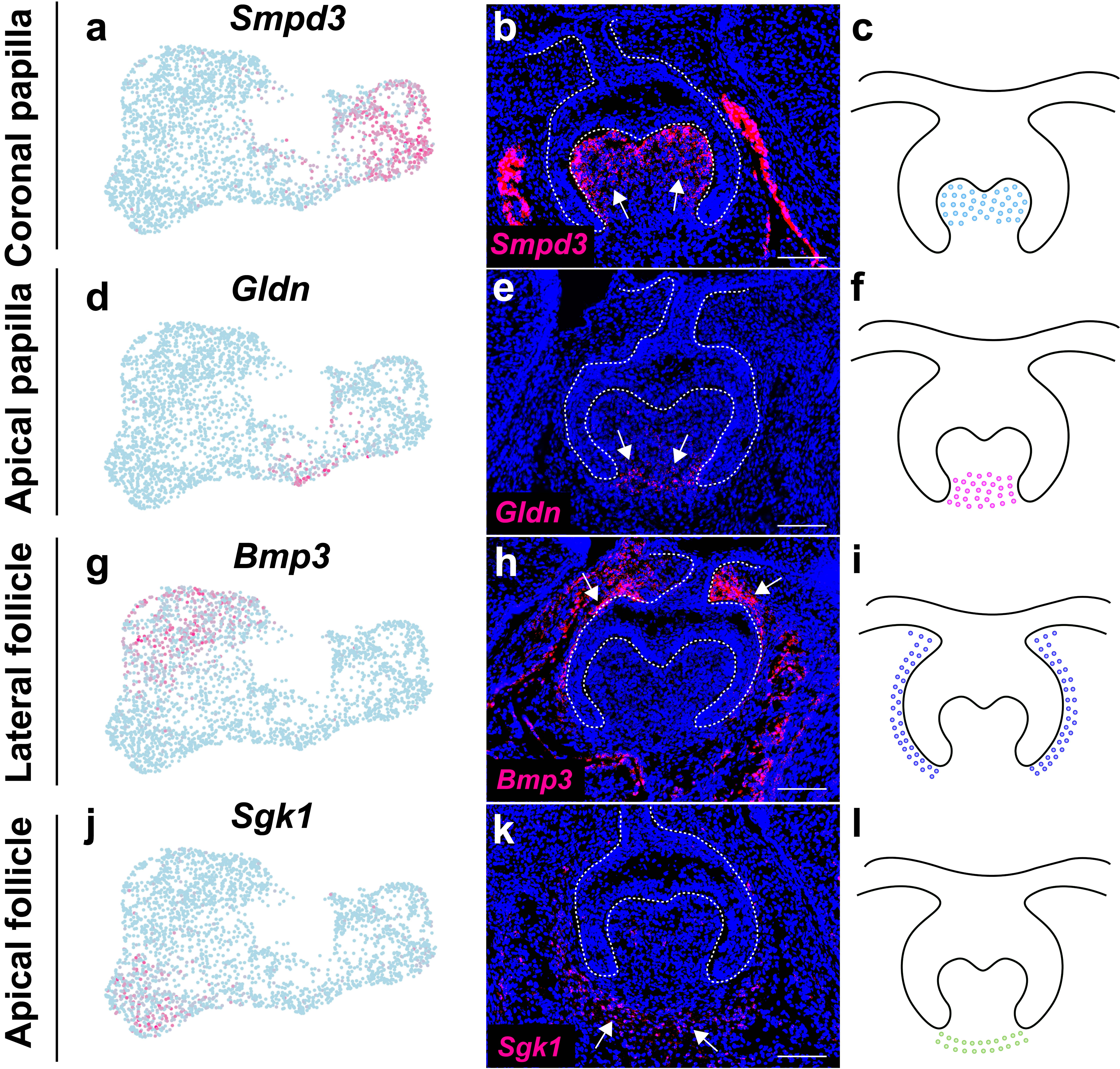
Coronal papilla
a
Smpd3
b
Smpd3
c
Apical papilla
d
Gldn
e
Gldn
f
Lateral follicle
g
Bmp3
h
Bmp3
i
Apical follicle
j
Sgk1
k
Sgk1
l

Supplementary Fig. 5 Cellular domain of the dental mesenchyme at E16.5. a, **b** Feature plot and RNAscope staining of *Smpd3* in the coronal papilla (n=3). **c** Cellular domain of the coronal papilla in the mouse molar at E16.5. **d**, **e** Feature plot and RNAscope staining of *Gldn* in the apical papilla of the mouse molar at E16.5 (n=3). **f** Cellular domain of the apical papilla in the mouse molar at E16.5. **g**, **h** Feature plot and RNAscope staining of *Bmp3* in the lateral follicle (n=3). **i** Cellular domain of the lateral follicle in the mouse molar at E16.5. **j**, **k** Feature plot and RNAscope staining of *Sgk1* in the apical follicle (n=3). **l** Cellular domain of the apical follicle in the mouse molar at E16.5. White dotted lines outline the dental epithelial cells in the mouse molar. White arrows point to the positive signals. Scale bars, 100μm.

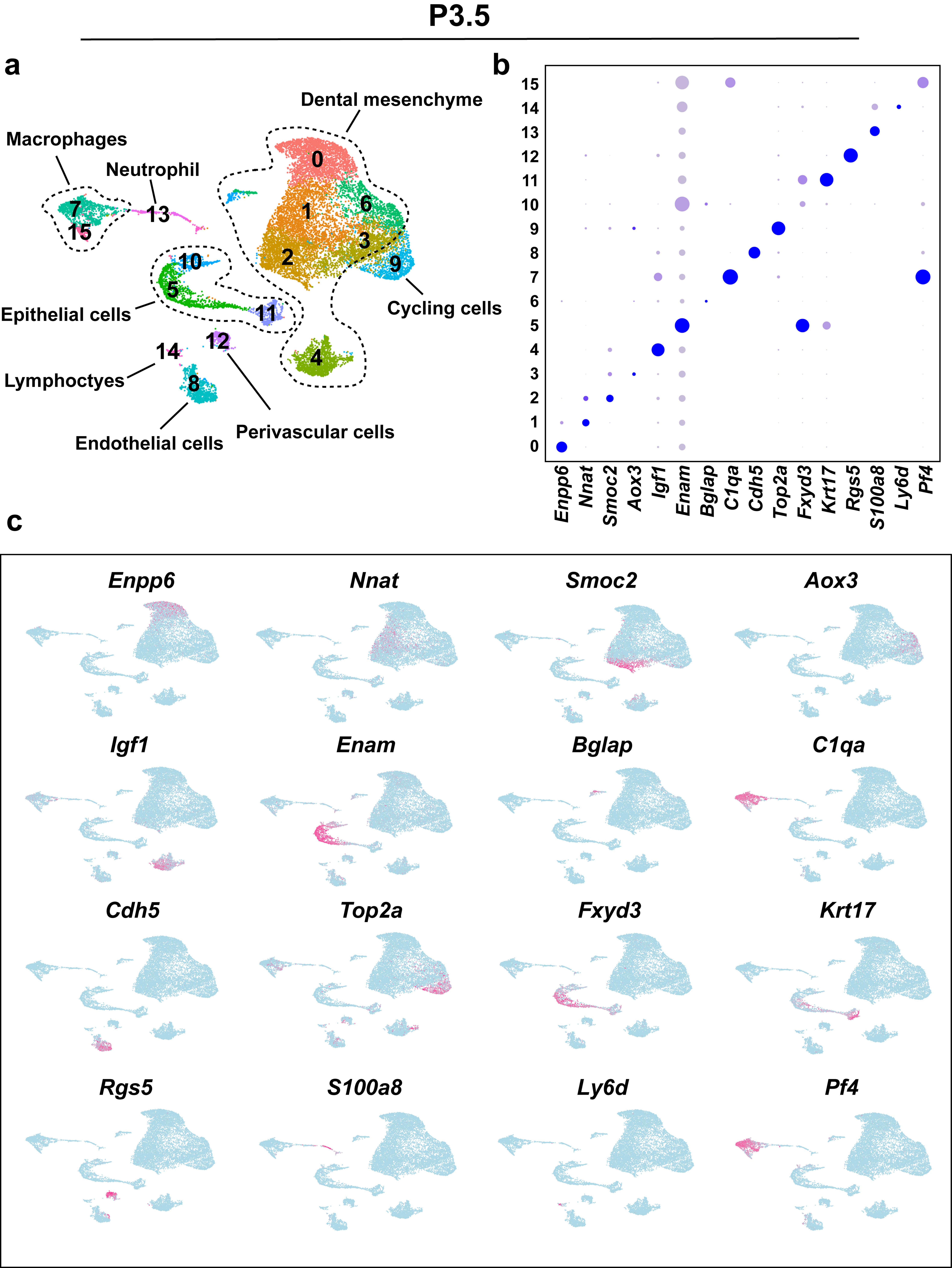
P3.5
a
Dental mesenchyme
Macrophages
Neutrophil
Epithelial cells
Cycling cells
Lymphoctyes
Endothelial cells
Perivascular cells
0
1
2
3
4
5
6
7
8
9
10
11
12
13
14
15
b
Enpp6
Nnat
Smoc2
Aox3
Igf1
Enam
Bglap
C1qa
Cdh5
Top2a
Fxyd3
Krt17
Rgs5
S100a8
Ly6d
Pf4
c
Enpp6
Nnat
Smoc2
Aox3
Igf1
Enam
Bglap
C1qa
Cdh5
Top2a
Fxyd3
Krt17
Rgs5
S100a8
Ly6d
Pf4

Supplementary Fig. 6 Cell populations in the mouse molar at P3.5. a UMAP plot of cell types in the mouse at P3.5. **b** Dotplot of signature genes in the cell populations shown in **a**. **c** Feature plot of markers genes shown in **b**.

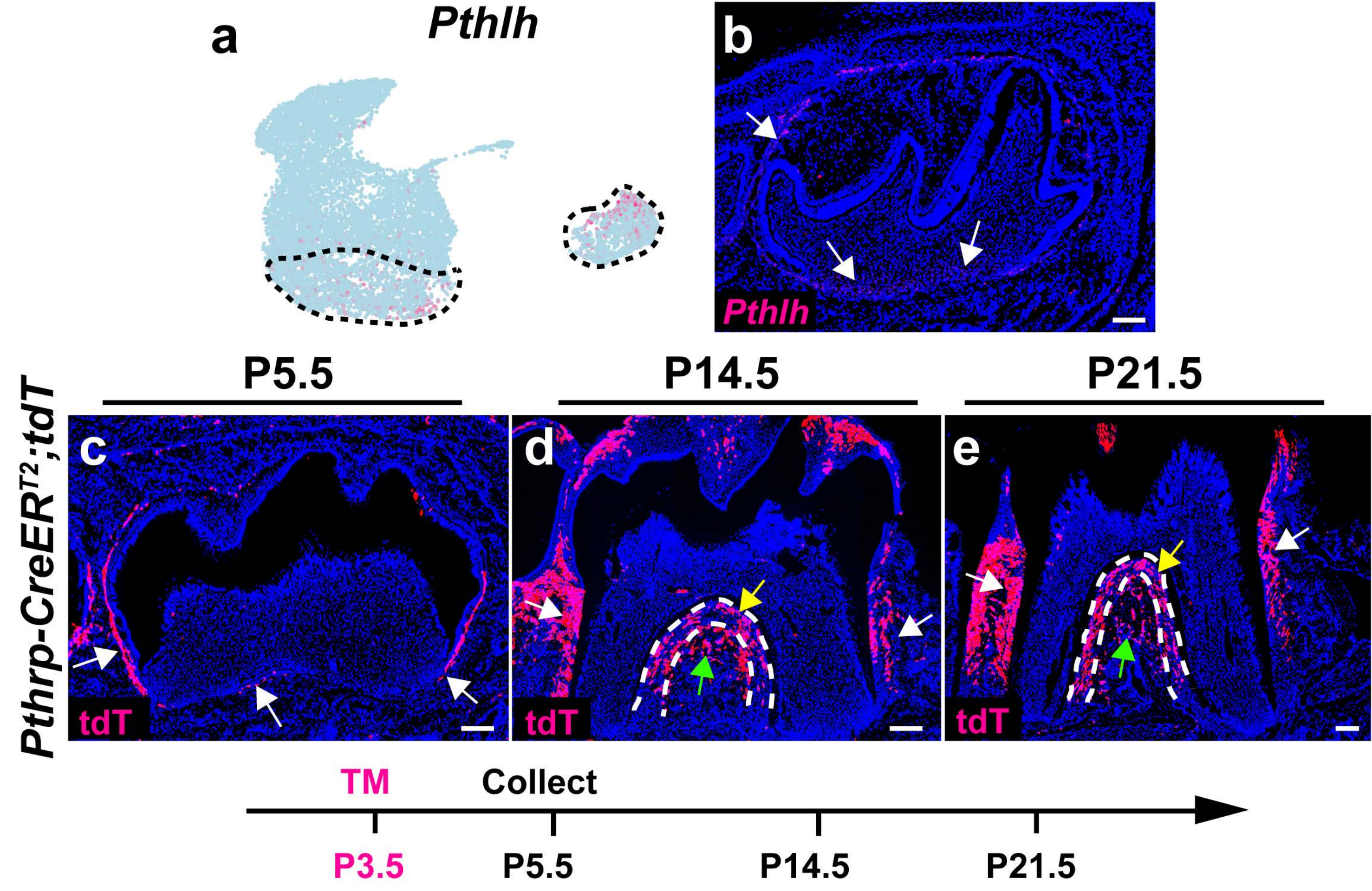

a
Pthlh
b
Pthlh
P5.5
P14.5
P21.5
Pthrp-CreER^{T2};tdT
c
d
e
tdT
tdT
tdT
TM
Collect
P3.5
P5.5
P14.5
P21.5

Supplementary Fig. 7 Pthrp+ cells contribute to the periodontal tissue in both root-forming and furcation regions. a, **b** Feature plot and RNAscope staining of *Pthlh* in the mouse molar at P3.5 (n=3). **c-e** Lineage tracing analysis of Pthrp+ cells in the mouse molar from P3.5 to P21.5 (n=3). Induction protocol is at the bottom of the figures. White dotted lines outline the PDL in the mouse molar. White arrows point to the positive signals. Yellow and green arrows in **d** and **e** point to the positive signals in PDL and alveolar bone in the root furcation region, respectively. Black dotted lines outline the cellular domains in the feature plots. Scale bars, 100μm.

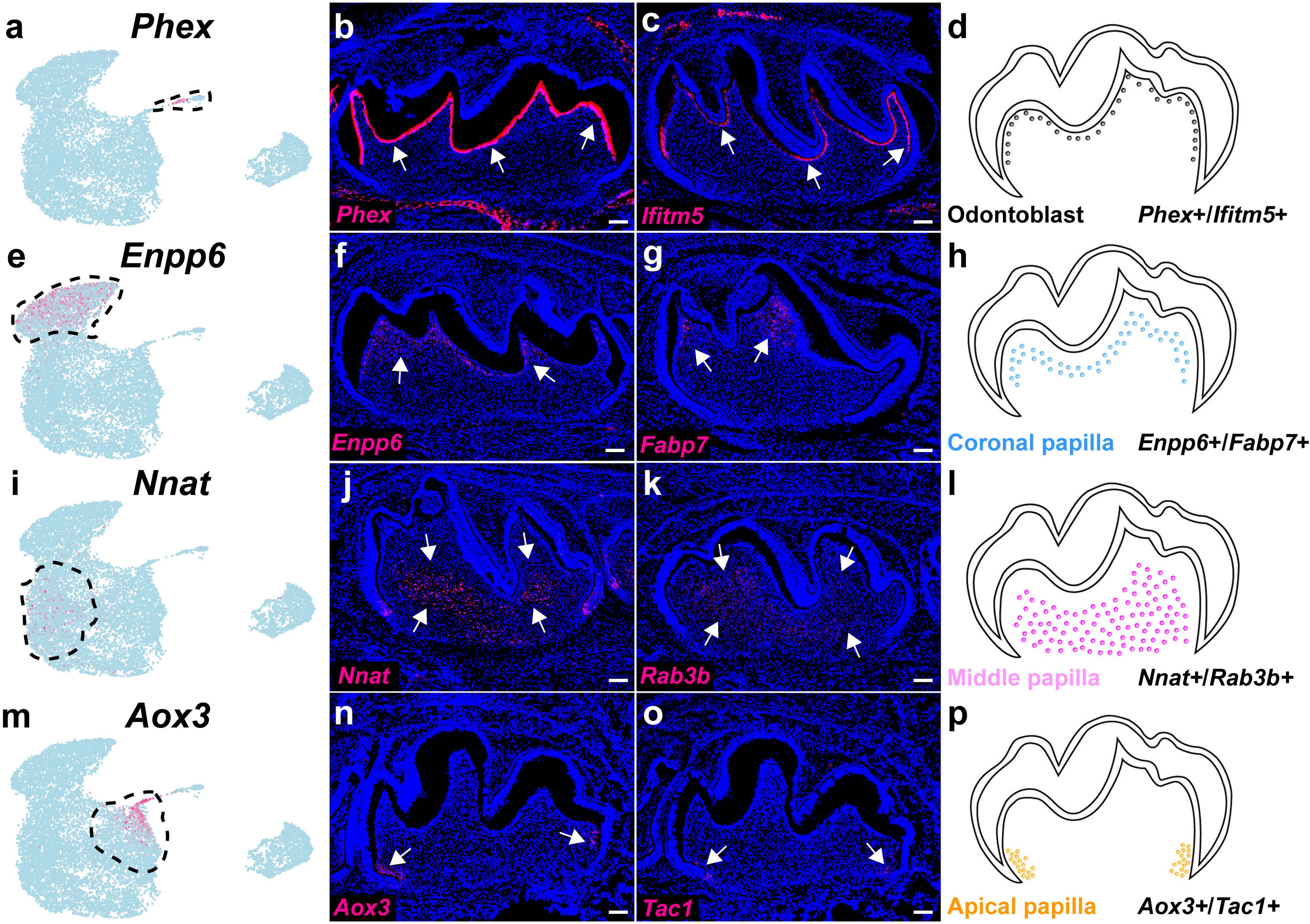
a
Phex
b
Phex
c
Ifitm5
d
Odontoblast
Phex+/Ifitm5+
e
Enpp6
f
Enpp6
g
Fabp7
h
Coronal papilla
Enpp6+/Fabp7+
i
Nnat
j
Nnat
k
Rab3b
l
Middle papilla
Nnat+/Rab3b+
m
Aox3
n
Aox3
o
Tac1
p
Apical papilla
Aox3+/Tac1+

Supplementary Fig. 8 Cellular domains in the dental papilla of the mouse molar at P3.5. a-c Feature plot and RNAscope staining of odontoblast markers in the mouse molar at P3.5 (n=3). **d** Schematic drawing of odontoblast domain in the mouse molar at P3.5. **e-g** Feature plot and RNAscope staining of coronal papilla markers in the mouse molar at P3.5 (n=3). **h** Schematic drawing of the coronal papilla domain in the mouse molar at P3.5. **i-k** Feature plot and RNAscope staining of middle papilla markers in the mouse molar at P3.5 (n=3). **l** Schematic drawing of the middle papilla domain in the mouse molar at P3.5. **m-o** Feature plot and RNAscope staining of apical papilla markers in the mouse molar at P3.5 (n=3). **p** Schematic drawing of the apical papilla domain in the mouse molar at P3.5. Black dotted lines outline the cellular domains in the feature plots. White arrows point to the positive signals. Scale bars, 100μm.

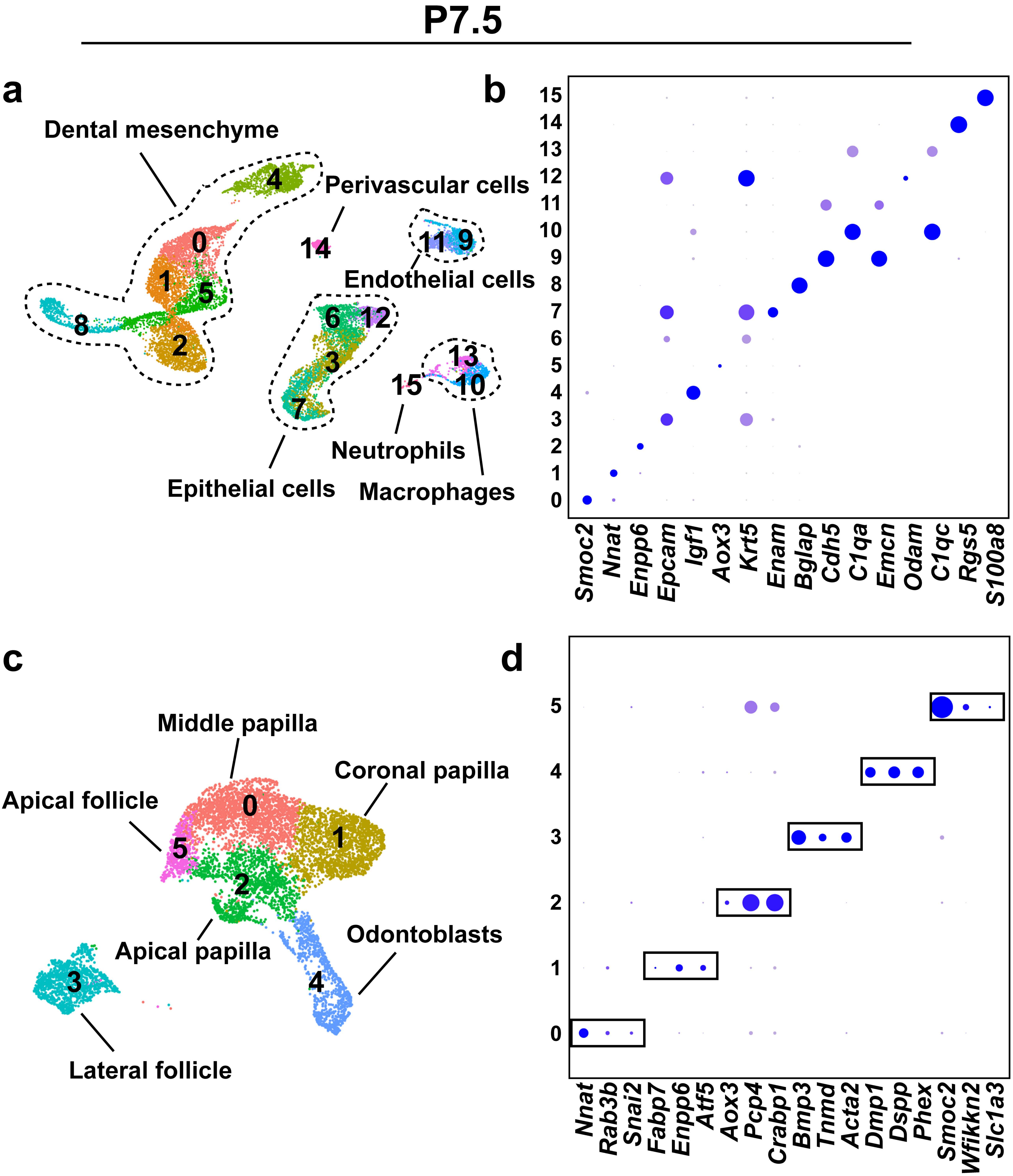
P7.5
a
Dental mesenchyme
Perivascular cells
Endothelial cells
Epithelial cells
Neutrophils
Macrophages
0
1
2
4
5
8
14
11
9
6
12
3
7
15
13
10
b
Smoc2
Nnat
Enpp6
Epcam
Igf1
Aox3
Krt5
Enam
Bglap
Cdh5
C1qa
Emcn
Odam
C1qc
Rgs5
S100a8
c
Middle papilla
Coronal papilla
Apical follicle
Apical papilla
Odontoblasts
Lateral follicle
d
Nnat
Rab3b
Snai2
Fabp7
Enpp6
Atf5
Aox3
Pcp4
Crabp1
Bmp3
Tnmd
Acta2
Dmp1
Dspp
Phex
Smoc2
Wfikkn2
Slc1a3

Supplementary Fig. 9 Cell populations in the mouse molar at P7.5. a UMAP plot of cell types in the mouse molar at P7.5. **b** Dotplot of signature genes of the cell populations shown in **a**. **c** UMAP plot of cell populations in the dental mesenchyme at P7.5. **d** Dotplot of signature genes of the cell populations shown in **c**.

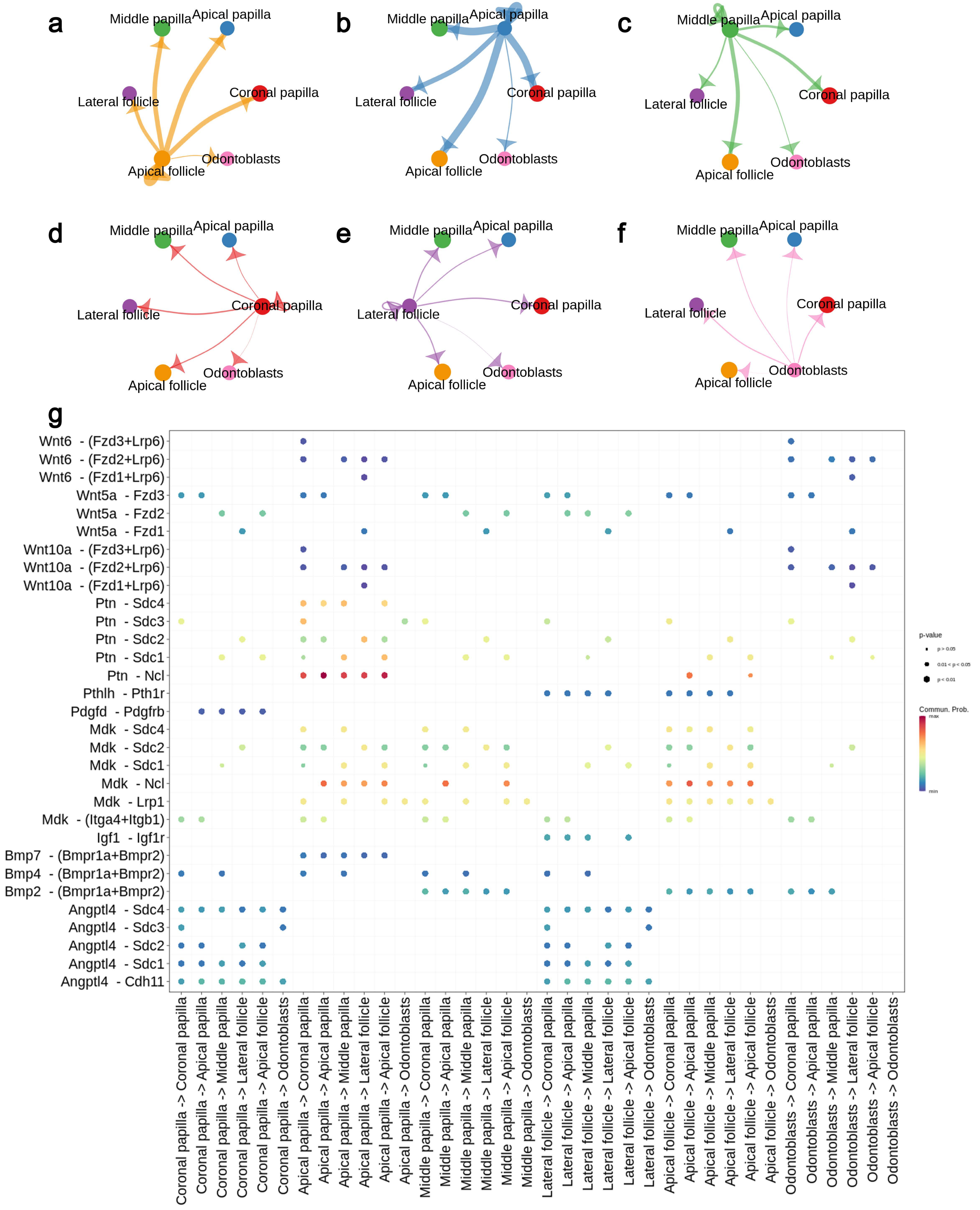
a
b
c
d
e
f
g
Middle papilla
Apical papilla
Lateral follicle
Coronal papilla
Apical follicle
Odontoblasts
Wnt6 - (Fzd3+Lrp6)
Wnt6 - (Fzd2+Lrp6)
Wnt6 - (Fzd1+Lrp6)
Wnt5a - Fzd3
Wnt5a - Fzd2
Wnt5a - Fzd1
Wnt10a - (Fzd3+Lrp6)
Wnt10a - (Fzd2+Lrp6)
Wnt10a - (Fzd1+Lrp6)
Ptn - Sdc4
Ptn - Sdc3
Ptn - Sdc2
Ptn - Sdc1
Ptn - Ncl
Pthlh - Pth1r
Pdgfd - Pdgfrb
Mdk - Sdc4
Mdk - Sdc2
Mdk - Sdc1
Mdk - Ncl
Mdk - Lrp1
Mdk - (Itga4+Itgb1)
Igf1 - Igf1r
Bmp7 - (Bmpr1a+Bmpr2)
Bmp4 - (Bmpr1a+Bmpr2)
Bmp2 - (Bmpr1a+Bmpr2)
Angptl4 - Sdc4
Angptl4 - Sdc3
Angptl4 - Sdc2
Angptl4 - Sdc1
Angptl4 - Cdh11
p-value
Commun. Prob.
Coronal papilla -> Coronal papilla
Coronal papilla -> Apical papilla
Coronal papilla -> Middle papilla
Coronal papilla -> Lateral follicle
Coronal papilla -> Apical follicle
Coronal papilla -> Odontoblasts
Apical papilla -> Coronal papilla
Apical papilla -> Apical papilla
Apical papilla -> Middle papilla
Apical papilla -> Lateral follicle
Apical papilla -> Apical follicle
Apical papilla -> Odontoblasts
Middle papilla -> Coronal papilla
Middle papilla -> Apical papilla
Middle papilla -> Middle papilla
Middle papilla -> Lateral follicle
Middle papilla -> Apical follicle
Middle papilla -> Odontoblasts
Lateral follicle -> Coronal papilla
Lateral follicle -> Apical papilla
Lateral follicle -> Middle papilla
Lateral follicle -> Lateral follicle
Lateral follicle -> Apical follicle
Lateral follicle -> Odontoblasts
Apical follicle -> Coronal papilla
Apical follicle -> Apical papilla
Apical follicle -> Middle papilla
Apical follicle -> Lateral follicle
Apical follicle -> Apical follicle
Apical follicle -> Odontoblasts
Odontoblasts -> Coronal papilla
Odontoblasts -> Apical papilla
Odontoblasts -> Middle papilla
Odontoblasts -> Lateral follicle
Odontoblasts -> Apical follicle
Odontoblasts -> Odontoblasts

Supplementary Fig. 10 Cell-cell interactions in the dental mesenchyme of the mouse molar at P3.5. a-f Interaction between cellular domains in the dental mesenchyme in the mouse molar at P3.5. **g** Ligand-receptor interactions in the cellular domains of the dental mesenchyme at P3.5. The significant interactions between two cell groups are identified using a permutation test. The interactions with p-value < 0.05 are considered significant.

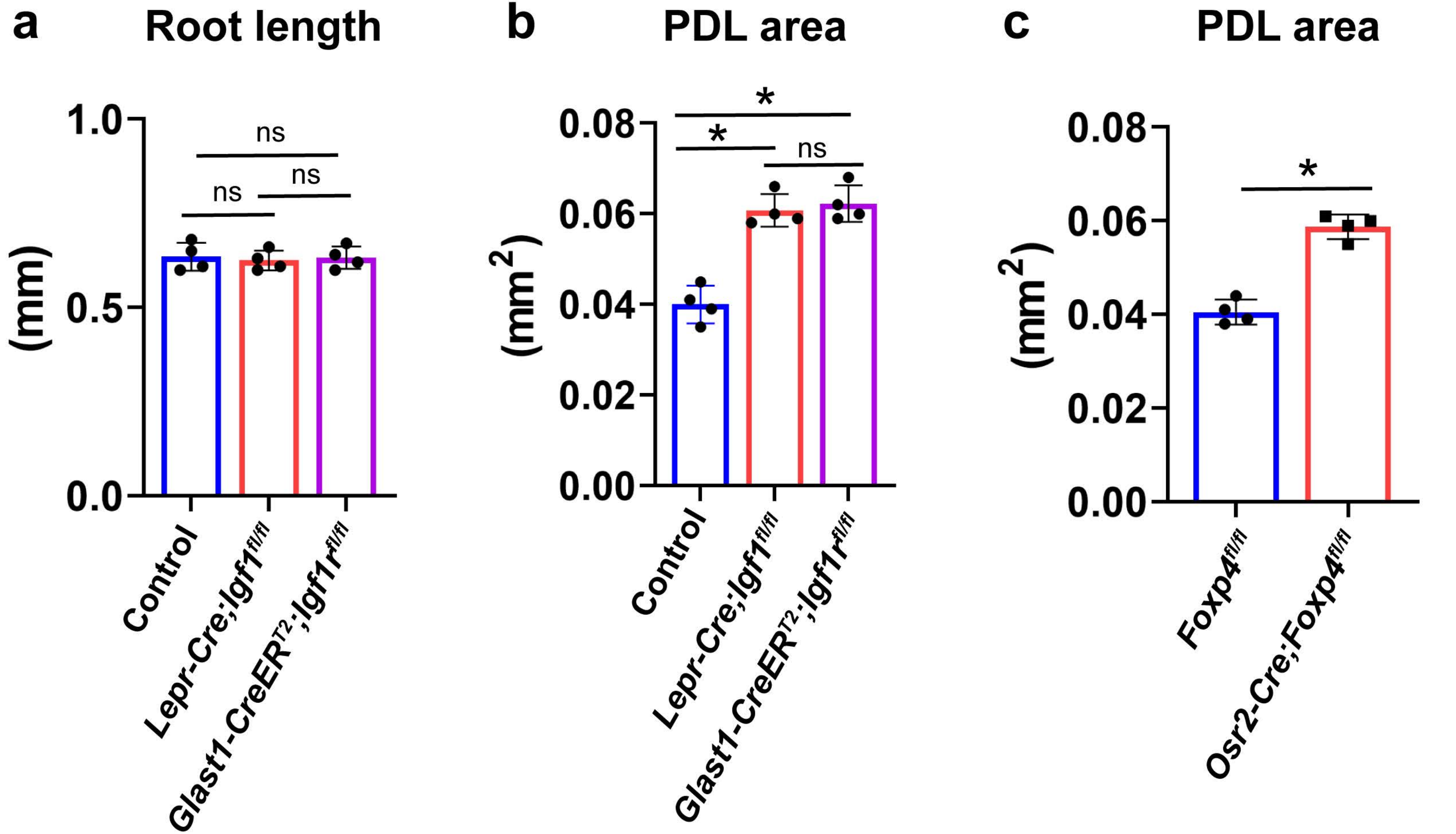

a
Root length
(mm)
1.0
0.5
0.0
ns
ns
ns
Control
Lepr-Cre;Igf1fl/fl
Glast1-CreERT2;Igf1rfl/fl
b
PDL area
(mm2)
0.08
0.06
0.04
0.02
0.00
*
*
ns
Control
Lepr-Cre;Igf1fl/fl
Glast1-CreERT2;Igf1rfl/fl
c
PDL area
(mm2)
0.08
0.06
0.04
0.02
0.00
*
Foxp4fl/fl
Osr2-Cre;Foxp4fl/fl

Supplementary Fig. 11 Quantitative analysis of root length and PDL area after disruption of Igf1 and Foxp4 signaling. a,b Quantitative analysis of root length and PDL area in control, *Lepr-Cre;Igf1*$^{fl/fl}$ and *Slc1a3-CreER*^{T2}*;Igf1r*$^{fl/fl}$ molars. **c** Quantitative analysis of PDL area in in *Foxp4*$^{fl/fl}$ and *Osr2-Cre;Foxp4*$^{fl/fl}$ molars. Data are presented as mean values ± SD. n=4 biologically independent samples. ns, no significant difference. p values are given for one-way ANOVA. *, $p < 0.0001$.

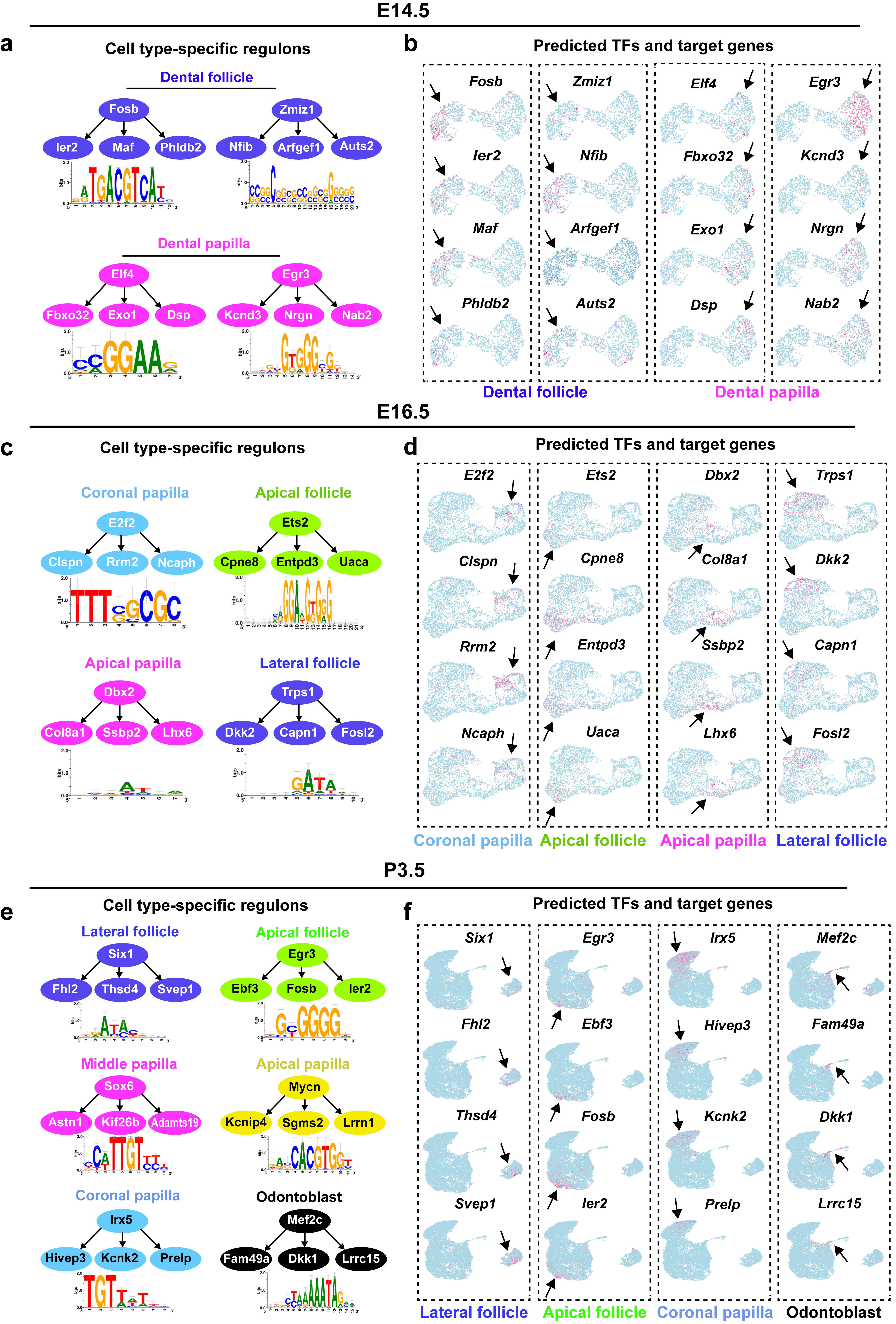

E14.5
a
Cell type-specific regulons
Dental follicle
Fosb
Ier2
Maf
Phldb2
Zmiz1
Nfib
Arfgef1
Auts2
Dental papilla
Elf4
Fbxo32
Exo1
Dsp
Egr3
Kcnd3
Nrgn
Nab2
b
Predicted TFs and target genes
Fosb
Ier2
Maf
Phldb2
Zmiz1
Nfib
Arfgef1
Auts2
Elf4
Fbxo32
Exo1
Dsp
Egr3
Kcnd3
Nrgn
Nab2
Dental follicle
Dental papilla
E16.5
c
Cell type-specific regulons
Coronal papilla
E2f2
Clspn
Rrm2
Ncaph
Apical follicle
Ets2
Cpne8
Entpd3
Uaca
Apical papilla
Dbx2
Col8a1
Ssbp2
Lhx6
Lateral follicle
Trps1
Dkk2
Capn1
Fosl2
d
Predicted TFs and target genes
E2f2
Clspn
Rrm2
Ncaph
Ets2
Cpne8
Entpd3
Uaca
Dbx2
Col8a1
Ssbp2
Lhx6
Trps1
Dkk2
Capn1
Fosl2
Coronal papilla
Apical follicle
Apical papilla
Lateral follicle
P3.5
e
Cell type-specific regulons
Lateral follicle
Six1
Fhl2
Thsd4
Svep1
Apical follicle
Egr3
Ebf3
Fosb
Ier2
Middle papilla
Sox6
Astn1
Kif26b
Adamts19
Apical papilla
Mycn
Kcnip4
Sgms2
Lrrn1
Coronal papilla
Irx5
Hivep3
Kcnk2
Prelp
Odontoblast
Mef2c
Fam49a
Dkk1
Lrrc15
f
Predicted TFs and target genes
Six1
Fhl2
Thsd4
Svep1
Egr3
Ebf3
Fosb
Ier2
Irx5
Hivep3
Kcnk2
Prelp
Mef2c
Fam49a
Dkk1
Lrrc15
Lateral follicle
Apical follicle
Coronal papilla
Odontoblast

Supplementary Fig. 12 Selected cell type-specific regulons in the dental mesenchyme. a Cell type-specific regulons highlighted in the dental mesenchyme at E14.5. **b** Feature plot of predicated TFs and corresponding target genes shown in **a**. **c** Cell type-specific regulons highlighted in the dental mesenchyme at E16.5. **d** Feature plot of predicated TFs and corresponding target genes shown in **c**. **e** Cell type-specific regulons highlighted in the dental mesenchyme at P3.5. **f** Feature plot of predicated TFs and corresponding target genes shown in **e**.
