# Supplementary material for: Spatiotemporal single-cell regulatory atlas reveals neural crest lineage diversification and cellular function during tooth morphogenesis
Source: Nat Commun. 2022 Aug 16;13:4803. doi: 10.1038/s41467-022-32490-y (PMC9381504; doi:10.1038/s41467-022-32490-y)
Supplement: Supplementary file 3 — Reporting Summary [file 41467_2022_32490_MOESM3_ESM.pdf]

nature portfolio

Corresponding author(s): Yang Chai

Last updated by author(s): Jul 22, 2022

Reporting Summary

Nature Portfolio wishes to improve the reproducibility of the work that we publish. This form provides structure for consistency and transparency in reporting. For further information on Nature Portfolio policies, see our Editorial Policies and the Editorial Policy Checklist.

Statistics

For all statistical analyses, confirm that the following items are present in the figure legend, table legend, main text, or Methods section.

n/a	Confirmed	
☐	☒	The exact sample size (*n*) for each experimental group/condition, given as a discrete number and unit of measurement
☒	☐	A statement on whether measurements were taken from distinct samples or whether the same sample was measured repeatedly
☐	☒	The statistical test(s) used AND whether they are one- or two-sided<br>*Only common tests should be described solely by name; describe more complex techniques in the Methods section.*
☒	☐	A description of all covariates tested
☒	☐	A description of any assumptions or corrections, such as tests of normality and adjustment for multiple comparisons
☐	☒	A full description of the statistical parameters including central tendency (e.g. means) or other basic estimates (e.g. regression coefficient) AND variation (e.g. standard deviation) or associated estimates of uncertainty (e.g. confidence intervals)
☐	☒	For null hypothesis testing, the test statistic (e.g. *F*, *t*, *r*) with confidence intervals, effect sizes, degrees of freedom and *P* value noted<br>*Give P values as exact values whenever suitable.*
☒	☐	For Bayesian analysis, information on the choice of priors and Markov chain Monte Carlo settings
☒	☐	For hierarchical and complex designs, identification of the appropriate level for tests and full reporting of outcomes
☒	☐	Estimates of effect sizes (e.g. Cohen's *d*, Pearson's *r*), indicating how they were calculated

Our web collection on statistics for biologists contains articles on many of the points above.

Software and code

Policy information about availability of computer code

Data collection	No software used for data collection
Data analysis	GraphPad Prism (version 9.0.0), ImageJ (version 1.53s), CellRanger (version 3.1.0), Seurat (version 4.0), SeuratWrappers (version 0.1.0), Monocle (version 3), GeneSwitches (version 0.1.0), Velocyto (version 0.17), CellChat (version 1.4.0), SCENIC (version 1.1.2), scVelo (version 0.2.4), Velocyto R package (version 0.6).

For manuscripts utilizing custom algorithms or software that are central to the research but not yet described in published literature, software must be made available to editors and reviewers. We strongly encourage code deposition in a community repository (e.g. GitHub). See the Nature Portfolio guidelines for submitting code & software for further information.

Data

Policy information about availability of data

All manuscripts must include a data availability statement. This statement should provide the following information, where applicable:

- Accession codes, unique identifiers, or web links for publicly available datasets
- A description of any restrictions on data availability
- For clinical datasets or third party data, please ensure that the statement adheres to our policy

All single-cell RNA-seq datasets are available through the GEO database GSE189381 (https://www.ncbi.nlm.nih.gov/geo/query/acc.cgi?acc=GSE189381) and FaceBase data repository (Record ID A-QVNG, doi: http://facebase.org)

Field-specific reporting

Please select the one below that is the best fit for your research. If you are not sure, read the appropriate sections before making your selection.

☒ Life sciences ☐ Behavioural & social sciences ☐ Ecological, evolutionary & environmental sciences

For a reference copy of the document with all sections, see nature.com/documents/nr-reporting-summary-flat.pdf

Life sciences study design

All studies must disclose on these points even when the disclosure is negative.

Sample size	Total amount of sequenced cells was 112,239 including different stages of the mouse molar samples. Sample size was sufficient to identify not only major populations, but also very small subpopulations in the dental mesenchyme. Data were consistent when compared to different samples analyzed in this study.
Data exclusions	Cells which didn't pass the quality check were excluded from the analysis in an unbiased way. Poor-quality cells with less than 200 genes and mitochondrial gene percentages > 50 (to take into account high mitochondrial content) were removed in this study.
Replication	All experiments were done in replicate. For scRNAseq, each sequenced library is the result of the pooling of at least 10 molar samples. Immunostaining, RNAscope staining and lineage tracing experiments were performed in three experimental replicates.
Randomization	Cells were isolated from mouse molars for the scRNA-seq. No specific selection during isolation was applied. For analysis of embryonic mouse molars, at least 8 embryos were randomly selected from the pregnant female at each stage. For analysis of postnatal mouse molars, at least 5 pups were selected randomly at each stage.
Blinding	All the bioinformatic parts of single cell clustering were made in fully unbiased manner. Outcomes were then analyzed by biologists. Blinding was not relevant for this study.

Reporting for specific materials, systems and methods

We require information from authors about some types of materials, experimental systems and methods used in many studies. Here, indicate whether each material, system or method listed is relevant to your study. If you are not sure if a list item applies to your research, read the appropriate section before selecting a response.

Materials & experimental systems

n/a	Involved in the study	
☐	☒	Antibodies
☒	☐	Eukaryotic cell lines
☒	☐	Palaeontology and archaeology
☐	☒	Animals and other organisms
☒	☐	Human research participants
☒	☐	Clinical data
☒	☐	Dual use research of concern

Methods

n/a	Involved in the study	
☒	☐	ChIP-seq
☒	☐	Flow cytometry
☒	☐	MRI-based neuroimaging

Antibodies

Antibodies used	Rabbit anti-Foxp4 (1:200, HPA007176, MilliporeSigma)<br>Rabbit anti-Periostin (1:100, ab14041, Abcam)<br>Rabbit anti-Ki67 (1:100, ab15580, Abcam)<br>Goat anti-Rabbit secondary antibodies (1:200, A-11011, Thermo Fisher Scientific)
Validation	Rabbit anti-Foxp4 Supplier webpage – proved to be working on immunohistochemical staining on human stomach glandular cells. https://www.sigmaaldrich.com/US/en/product/sigma/hpa007176<br>Rabbit anti-Periostin Supplier webpage – proved to be working on immunohistochemistry staining of formalin-fixed paraffin-embedded human normal skin tissue. https://www.abcam.com/periostin-antibody-ab14041.html#lb<br>Rabbit anti-Ki67 Supplier webpage – proved to be working on immunohistochemistry staining of mouse tumour tissue sections. https://www.abcam.com/ki67-antibody-ab15580.html#lb

Animals and other organisms

Policy information about studies involving animals; ARRIVE guidelines recommended for reporting animal research

Laboratory animals	mouse line Fgf3-CreERT2 – Both males and females. Age between 2-4 months.<br>mouse line Foxp4flox/flox – Both males and females. Age between 2-4 months.

mouse line Igf1flox/flox – Both males and females. Age between 2-4 months.
mouse line Igf1rflox/flox – Both males and females. Age between 2-4 months.
mouse line Lepr-Cre – Both males and females. Age between 2-4 months.
mouse line Lhx6–CreERT2– Both males and females. Age between 2-4 months.
mouse line Osr2-Cre – Both males and females. Age between 2-4 months.
mouse line Pax9-CreERT2 – Both males and females. Age between 2-4 months.
mouse line Pthrp-CreERT2 – Both males and females. Age between 2-4 months.
mouse line Rosa26<fstdTomato>– Both males and females. Age between 2-4 months.
mouse line Slc1a3-CreERT2 – Both males and females. Age between 2-4 months.

All mice were kept under SPF conditions in 12 light/12 dark cycle, 18-23°C and 40-60% humidity.

Wild animals

The study did not involve wild animals

Field-collected samples

The study did not involve samples collected from the field

Ethics oversight

All mouse procedures were approved by the Department of Animal Resources and Institutional Animal Care and Use Committee of the University of Southern California (protocol 20299).

Note that full information on the approval of the study protocol must also be provided in the manuscript.
